# Nature-Based Feasibility Intervention to Influence Mitigation Strategies for Perceived Stress

**DOI:** 10.3390/ijerph191912277

**Published:** 2022-09-27

**Authors:** Amber L. Vermeesch, Alessandra Coro, Kira Mattes, Dylan Ostendorff, Erica Timko Olson, Layla Garrigues

**Affiliations:** 1 Department of Family and Community Nursing, School of Nursing, University of North Carolina Greensboro, Greensboro, NC 27402, USA; 2 School of Nursing & Health Innovations, University of Portland, Portland, OR 97203, USA; 3 School of Nursing, University of Minnesota, Minneapolis, MN 55455, USA

**Keywords:** nature-based intervention, wellness, health promotion, nature, perceived stress

## Abstract

Burnout, compassion fatigue, and perceived stress among undergraduate nursing students are significant factors leading to a poorer quality of life, decreased job satisfaction, and adversely impact patient outcomes. Burnout among undergraduate nursing students is a critical individual and workforce issue with contributing factors including a relentless schedule, academically challenging and rigorous programs, pressure to perform, and the completion of clinical hours caring for patients. This paper describes our feasibility study of a nature-based intervention (NBI) to reduce perceived stress and quality of life as it relates to burnout, and compassion fatigue. Quantitative data was collected through demographics, surveys, and electronic sensor data. The project’s aim was to determine the feasibility of NBI monitored by NatureDose^TM^ to decrease perceived stress burnout and compassion fatigue among undergraduate nursing students.

## 1. Introduction

Burnout, compassion fatigue, and perceived stress among undergraduate nursing students are significant factors which can lead to a poorer quality of life, decreased job satisfaction, and adversely impact patient outcomes [1,2]. Burnout is defined as an occupational phenomenon by the World Health Organization (WHO). WHO defines burnout as a syndrome that is caused by chronic stress in the workplace. It includes feelings of exhaustion, cynicism, negativism, and reduced efficacy [3]. Compassion fatigue can include similar feelings and effects, but unlike burnout, compassion fatigue is caused by one significant exposure in caring for someone going through a traumatic event, or multiple exposures of caring for others going through traumatic events. Compassion fatigue can come on more quickly than burnout, and if caught early, can be treated more effectively as well [4]. While perceived stress can be defined as the level at which events are considered stressful, unpredictable and uncontrollable [5]. Sleep levels are considered a reliable way to look at responses to stressors [6].

Burnout among undergraduate nursing students is a critical individual and workforce issue with contributing factors including a relentless schedule, academically challenging and rigorous programs, pressure to perform, and the completion of clinical hours caring for patients. The pivot to online and hybrid learning during the COVID-19 pandemic compounded these factors [7,8] Moreover, personal obligations to family and work, sleep disturbances, increased stress levels, complicated by isolation from peers due to the pandemic, contributed to the exponential increase in burnout among undergraduate nursing students.

Nature-based interventions (NBI) have been shown to have significant impacts on both physical health, and mental health and well-being. The physical health benefits of NBI that support decreased stress levels include decreased heart rate, blood pressure, and cortisol levels [9]. Several studies have found mental health benefits such as decreased depression, anxiety, and anger when spending time in nature [10,11,12,13]. Additionally, there is emerging evidence of the benefits of exposure to urban greenspaces. Accessible urban greenspaces can promote an increase in physical activity, increased time outdoors, that result in positive mental health outcomes [14]. Predictors of lower levels of stress include accessible greenspace, higher tree density, and increased numbers of greenspaces in neighborhoods [15,16]. This paper describes a feasibility study of a NBI to reduce perceived stress, burnout, and compassion fatigue among undergraduate nursing students. The purpose of this pilot study was to assess feasibility of a nature-based intervention using electronic sensors and an application, NatureDose™, that sent updates to the intervention group as they go into nature.

### Background

The recommended amount of exposure to natural environments, to maximizes overall wellbeing, is 120 min per week. [17] Even as little as ten minutes of exposure to nature has been shown to have a significantly positive impact on psychological well-being [18]. Enhancing wellbeing is an essential component to mitigating perceived stress and NBIs have been successfully implemented worldwide for a multitude of health benefits including reduction in perceived stress [19]. Moreover, exposure to nature is easily accessible and free for most people [20], and is an important self-care strategy providing benefits far beyond the benefits of physical activity [21]. Yet, there is a gap regarding studies investigating the feasibility of NBI among undergraduate nursing students.

## 2. Materials and Methods

Study procedures for this feasibility study were reviewed and approved by a university Institutional Review Board. Researchers took steps to preserve the confidentiality of study participants and the data collected by using participant identifiers encrypted and substituted with codes for data analysis. Upper division undergraduate nursing students, from a private university and public community college in the Pacific Northwest, were recruited to participate through the dissemination of flyers, announcements at university Student Nurses Association meetings, word of mouth, and direct introductory emails. Fifty-nine participants consented (intervention *n* = 28 and control *n* = 31)—all from the private university.

Inclusion criteria included the following:

o Upper division undergraduate nursing students
o Ability to read, write, and speak English;
o At least 18 years-old;
o No serious health conditions that could affect their ability to be in nature;
o Willingness to wear an electronic sensor continuously for four weeks, including sleep, excluding bathing and device recharging time;

Access to a smartphone and able to download the NatureDose™ app After screening participants for eligibility, participants met the researchers, an in-person, meeting time was arranged for each participant to receive their sensor and register with the study. Registration included: an introduction to the study, a written information sheet that provided a description of the study and procedures, s signing an electronic consent form and completing an online questionnaire via Qualtrics. Before the questionnaire administration portion of the survey began, participants were provided with an electronic information sheet. A pre-randomization Qualtrics survey was provided to participants which included a signature box for consent prior to receiving their electronic sensor and partaking in the study. The information sheet provided a description of the study and procedures. By submitting a signature via the signature box on the pre-randomization Qualtrics survey, consent was implied. Participants were also given the opportunity to ask questions about the study which were answered by the research team.

**Scheme 1:**
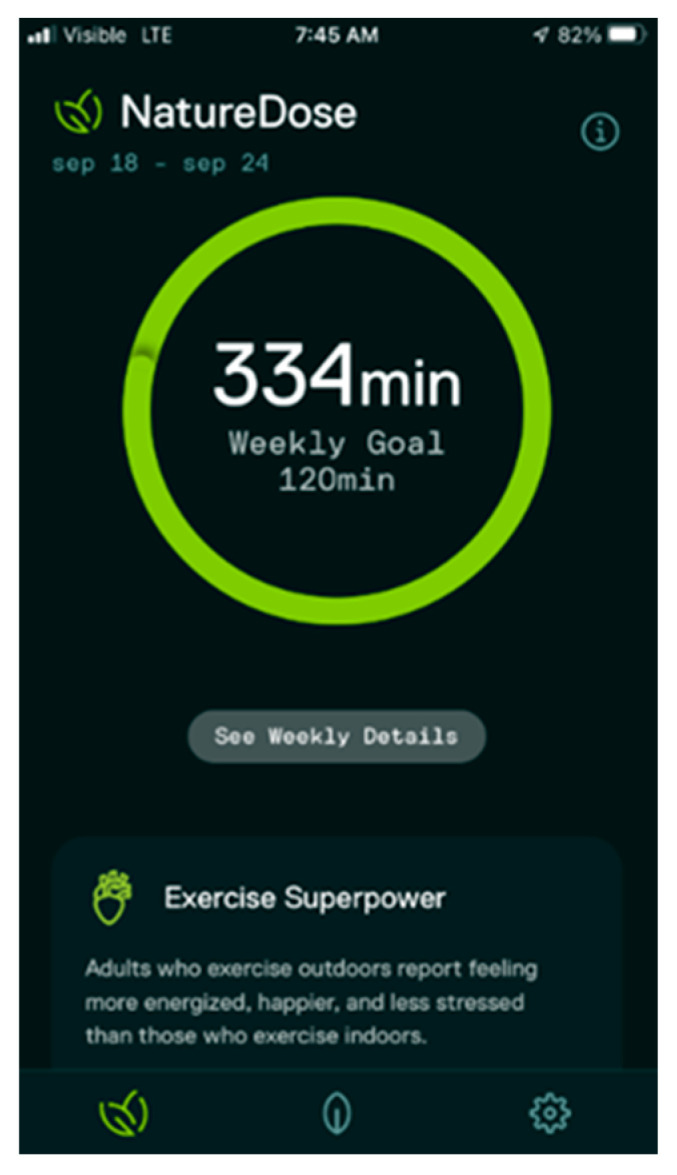
NatureDose Weekly Goal. Source: NatureQuant (www.naturequant.com)-2022.

**Scheme 2:**
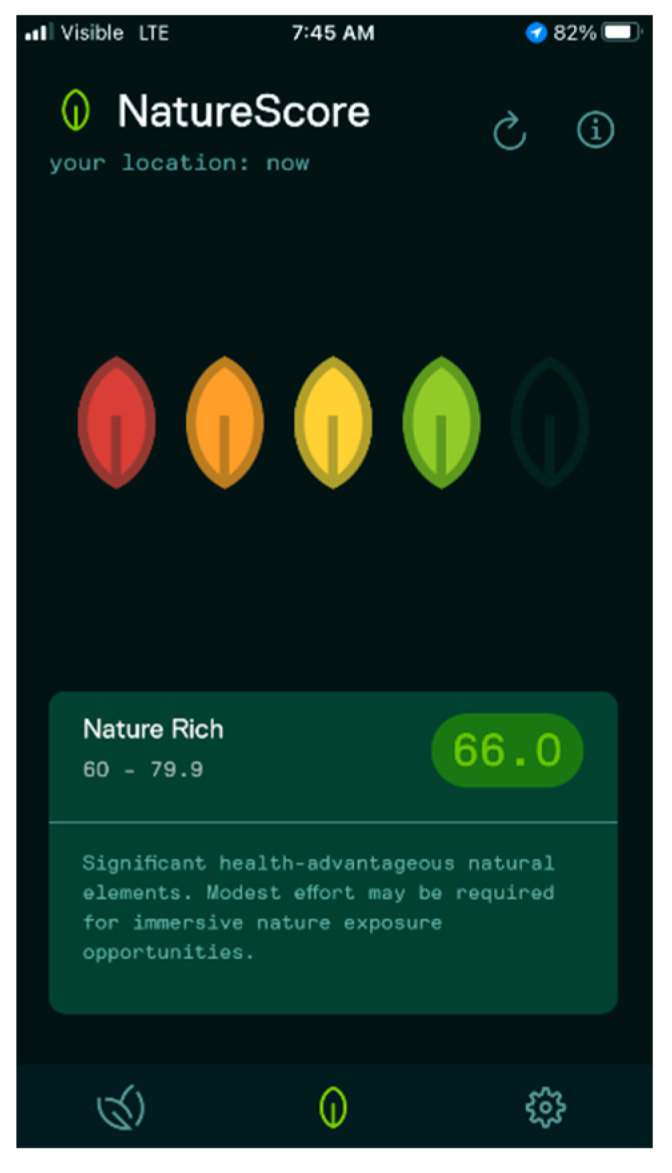
NatureDose Score. Source: NatureQuant (www.naturequant.com)-2022.

Participants were randomly divided into two groups: intervention and control. The intervention group downloaded the NatureDose™ app onto their individual self-provided smartphone. The control group did not download the NatureDose™ app. The goal for time outdoors was set, within the app, at 120 min per week and the participants received a notification when they achieved their goal of 120 min a week (see Scheme 1). If they did not achieve their goal, they did not receive a notification. The notification was a visual pop-up screen with a pictographic image. The app also showed a NatureScore (see Scheme 2) with the quality of nature exposure the intervention participants were engaged in as depicted by pictographic leaves (more leaves indicates higher quality exposure to nature) along with a written message encouraging participants to continue to get out in nature [22]. The participants could access the app and see their current minutes in nature for the week. The time reset each Sunday. Both the intervention and control groups were instructed to go about their lives as usual.

Participants were randomly placed into the intervention (*n* = 28) or control group (*n* = 31). For two participants, initially randomized to the intervention group, the NatureDose™ app would not function on their phones so they were transferred to the control group.

**Figure 1:**
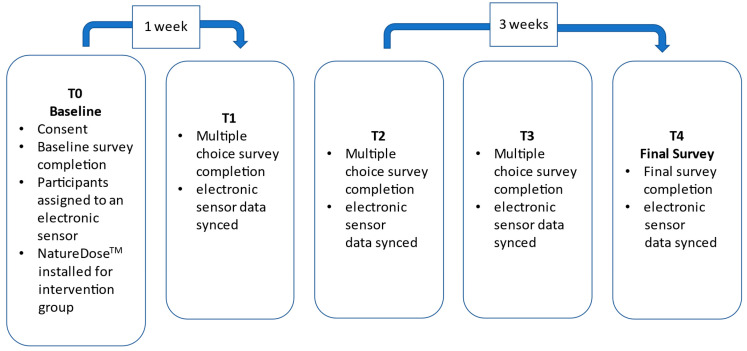
Data collection timeframe.

Data were collected five times (See Figure 1):

T0 = Pre-randomization, surveys, and NatureDose^TM^, the electronic sensors were provided to participants but no data was gathered at T0

T1 = Week 1 Surveys, and NatureDose^TM^, electronic sensor

T2 = Week 2 Surveys, and NatureDose^TM^, electronic sensor

T3 = Week 3 Surveys, and NatureDose^TM^, electronic sensor

T4 = Week 4 Surveys, and NatureDose^TM^ electronic sensor

### 2.1. Instruments

Instruments used were demographics including age range (i.e., 18–20, 21–25, 26–30, 31–35, >35), the Perceived Stress Scale (PSS), Professional Quality of Life Measure (ProQOL), WHO-5 Well-Being Index (WHO-5), and identified gender, and sensor data. All surveys were completed online via Qualtrics. Participants wore the Inspire 2 Fitbit for four weeks continuously. Exceptions were made for showering and charging the electronic sensor. The intervention group used the NatureDose™ app, downloaded to their phones, to monitor their time in nature

Perceived Stress Scale (PSS). PSS has been used extensively to measure self-reported stress and has demonstrated a reliability of alpha =0.78, and validity that correlates in a predicted way with other measures of stress including the Job Responsibilities Scale and Life Events Scales [23]. Items include: In the last month, how often have you been upset because of something that happened unexpectedly?, and In the last month, how often have you felt nervous and stressed?

Professional Quality of Life (ProQOL). The ProQOL is the most used and validated tool to measure the incidences of secondary trauma in nurses which is linked to compassion fatigue and is a sign of being human, not of failing [24]. The ProQOL 5 is easy to use and has been used individually and in groups, as well as in person and online [25]. The psychometrics of ProQOL include reliability of alpha =0.88 and validity with inter-scale correlations showing 2% shared variance (r = −0.23; co-σ = 5%; *n* = 1187) with secondary traumatic stress and 5% shared variance (r = −0.14; co-σ = 2%; *n* = 1187) with Burnout [24]. Items include: I get satisfaction from being able to [help] people and I think that I might have been affected by the traumatic stress of those I [help].

WHO-5 Well-Being Index (WHO-5). This index is a self-reported measure of current mental wellbeing with five questions on a Likert Scale with an adequate validity in measuring research outcomes [26]. The WHO-5 has been evaluated in multiple settings and disease processes for construct validity and has been determined that total WHO-5 score is a sufficient statistic [26]. Examples of questions include, “I have felt cheerful in good spirits” and “I have felt calm and relaxed” [27].

Electronic sensor data. Electronic sensor data via the Inspire 2 Fitbit included heart rate, sleep metrics, and daily activity measured in steps. Data from the electronic sensors was downloaded into a secure platform accessible to the PI, CO-PI, and student research assistants.

NatureDose™. This app was developed and owned NatureQuant. NatureDose™ is a personalized nature mobile application system that monitors an individual’s aggregate time inside, outside—beyond 10 feet of a structural boundary, and exposure to nature [22] When the user is outdoors, not in a designated natural area (park, wilderness, river, etc.), the app relies on the proprietary NatureScore location system to determine partial nature exposure. NatureScore™ measures the amount and quality of natural elements of any location using a patent-pending system. For each location, NatureQuant analyzes and blends various data sets and processed information within a given radius, including satellite infrared measurements, geographic information system (GIS) and land classifications, park data and features, tree canopies, air, noise, and light pollutions, and computer vision elements (aerial and street images). This results in a nature score that ranges from 0–100 with zero being largely a built environment and 100 being largely a natural environment, uniform distribution results in a score of 50. Scores of 0–19.9 indicates nature deficit, 20–39.9 indicates moderate to low density of health supporting natural elements, 40–59.9 indicates a balance of the built environment and health supporting natural elements, 60–79.9 indicates significant health advantageous natural elements and 80–100 indicates abundant health benefits and nature exposure [28].

### 2.2. Data Analysis

Quantitative data was analyzed to address the specified aims:

- Aim 1: Examine the feasibility to recruit, randomize, and retain subjects, and collect data on clinical outcomes of interest.
- Aim 2: Examine the feasibility to monitor exposure, quantity, and quality of time spent in natur from the NatureDose™ app and physiologic data from an electronic sensor.

Data from surveys, electronic sensors (i.e., Inspire 2 Fitbits), and NatureDose™ were compared to the control group. Quantitative results were analyzed using descriptive, boxplots and longitudinal mixed-effects modeling. Random effects were specified to account for the correlation of repeated measures on the same individuals [29] (Fitzmaurice, Laird, & Ware, 2011). Intervention group (Intervention (I) vs. Control (C)), week, and the interaction of group × week were specified as fixed effects. Of particular interest was this interaction, which if significant would mean that the I vs. C differences depend upon week whereas a non-significant effect would mean I vs. C differences do not significantly change across weeks (i.e., from baseline) [29]. Because model assumptions were not met for ProQOL scores, negative binomial mixed-effects modeling was performed which was robust to skewness of these scores. Negative binomial modeling has been previously used as an alternative to linear modeling for scale scores when assumptions not satisfied due to skewness/non-normality [30].

To accurately assess overall sleep, sleep times were averaged throughout the week, and then were compared to the results of in each participant’s Perceived Stress Scale, Professional Quality of Life, and the WHO-5 Well-Being Index.

## 3. Results

### 3.1. Demographic Data

The participants were junior and senior nursing students recruited from the private university in the Pacific Northwest. About 20% of the participants were between the ages of 18–20 (*n* = 12) and the majority of participants were between the ages of 21–25 (*n* = 47). The control group had seven participants between the ages of 18–20, while the Intervention group had five participants between the ages of 18–20.

The ratio of females to males was consistent with the current demographics of the nursing profession. A little more than 20% of the participants identified as male (*n* = 13) and all other participants identified as female (*n* = 46). The control group had seven participants identify as male and 24 identify as female while in the intervention group, six identified as male and 22 identified as female.

### 3.2. Recruitment, Randomization, and Retention Data

Recruitment took place on 2 college campuses with final participant being from only one campus. The number of participants sought was the study was 60 and 59 were consented with a 98.33% of the recruitment goal reached. All participants were willing to be randomized into either the control or intervention group. Fifty-nine participants completed the initial survey and 42 completed the final survey (control *n* = 20 and intervention *n* = 22), a final response rate of 71%.

**Figure 2:**
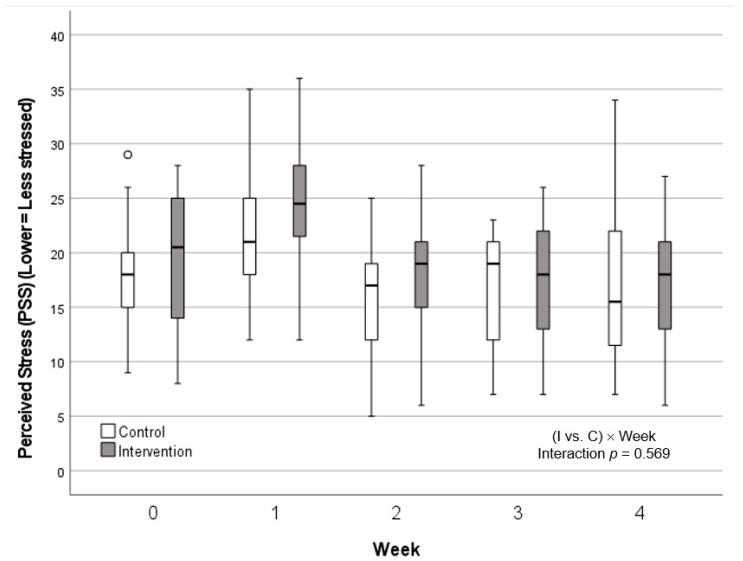
Perceived stress scores over time by group.

Survey Data Figure 2 gives the boxplots by group over time for perceived stress scores. Here, the inter-action of Intervention (I) versus Control (C) and week was not significant (*p* = 0.569).

**Figure 3:**
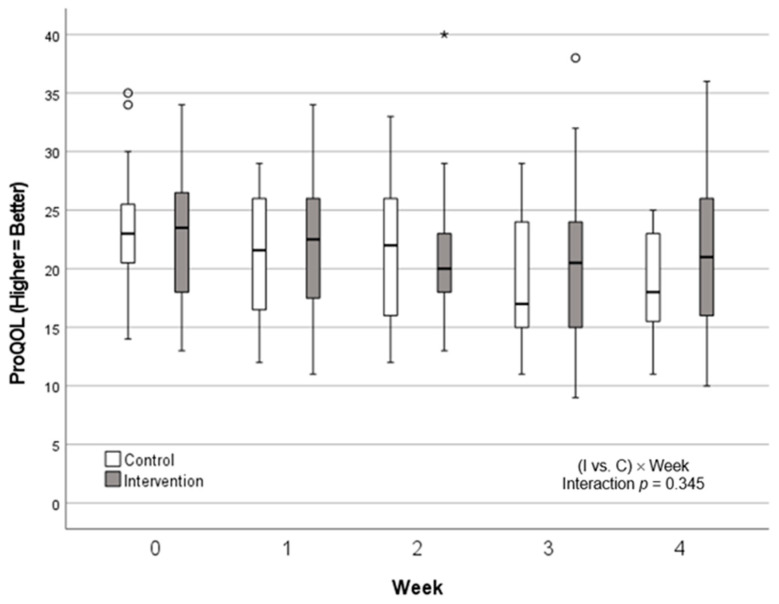
Professional quality of life (PROQOL) scores over time by group. * *p* < 0.05.

For professional quality of life, boxplots of the PROQOL scores over time are given in Figure 3. There was an extreme outlying score in the intervention group during week 2, indicated by the asterisk in Figure 3. However, again the interaction of Intervention (I) versus Control (C) and week was not significant (*p* = 0.345).

**Figure 4:**
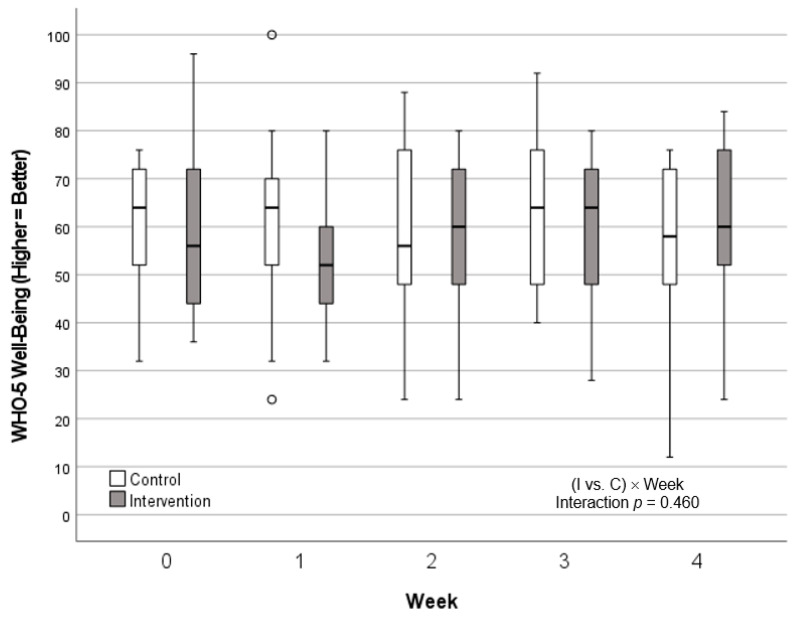
WHO Well-Being Index scores over time by group.

For the WHO Well-being index scores, boxplots in Figure 4 revealed minor differences between groups over time that were not statistically significant different (interaction I vs. C × week *p* = 0.460).

### 3.3. NatureDose™ Data

Participants consented to keep their phones with them will outside their primary residence. This resulted in data being collected during all four weeks of the study. Throughout the course of the study, participants spend an average of 25.1 min a day, and 179 min in nature a week with an average nature score of 58.74. Almost 53% of participants spent at an average of least 120 *min* in nature per week with incomplete data from 3 individuals

### 3.4. Sensor Data

Fifty-six of 59 participants (95%) had heart rate data available from the electronic sensors. Of these 56, 100% had electronic sensor data for Week 1, 98% for Week 2, 96% for Week 3, and 88% for Week 4. For electronic sensor-based steps, the 59 participants had at least 25 days of steps out of 28 on average. Given these findings we find that using electronic sensor for this population was indeed feasible. Table 1 provides the averages of the measures by group over time. There were no significant week-to-week differences for heart rate (HR), steps, or hours of sleep (all *p* ≥ 0.05).

## 4. Discussion

The purpose of this pilot study was to assess feasibility of a nature-based intervention using electronic sensors and an application, NatureDose™. Recruitment efforts were successful with a 98% of the target reached, however there was an attrition rate of 29%. This was possibly due the completion of the study coinciding with final semester exams. It is recommended to consent and additional 10% and be diligent of the university schedule and student availability at specified times during the school year. Participants were willing to participate in randomization, knowing they would not receive the intervention. The intervention could be offered to the control group following completion of the research study. Data collection proved challenging for some of the participants’ survey data. A detailed orientation to the data collection platform with ongoing support as needed may be beneficial for future studies. The attrition rate steadily increased over the course of the study; additional incentives may be beneficial in future studies.

Stress levels and heart rate are positively correlated, when increased stress is felt, it results in a higher heart rate [31]. Heart rate, a physiologic proxy for stress, were captured by the electronic sensor [32] Additionally, consideration was made on the technology’s ability to properly test for heart rate. Due to the green light that is used on the Inspire 2 Fitbit to test for heart rate, there is lower reliability among persons with darker skin tones. The wavelengths of the green light most accurately test heart rate for lighter skin tones, and thus these fluctuations were considered during data analysis [33]

Previous studies have alluded to higher sleep quality and quantity being positively correlated with morning happiness and negatively correlated with morning stress. Additionally, adolescents or young adults do not typically follow uniform sleep schedules; their sleep times often depend on the time of the week and their course or workload. [34]. The NatureDose™ was able be downloaded on all but two participants in the intervention group, they were moved to the control group. This should be discussed with the app developers before use in future studies. The app was able to accurately monitor participants time and quality of nature in their location. The data was easy for the researchers to view, download, and analyze.

This will help inform future studies developing standards for a recommendation of having at least 120 min per a week of nature exposure for undergraduate nursing students to decrease their stress and improve quality of life. It was difficult to enforce continuous wearing of electronic sensors, along with ensuring participants continued to sync their data with electronic sensor. Participants took off the electronic sensors for varying lengths of time (e.g., for bathing), and each participant had their own limitations of when they would and would not wear the electronic sensors. For example, some participants felt discomfort with wearing the electronic sensors during sleep, creating inconsistencies on what data was collected on each participant. Despite these challenges, 100% participants wore their electronic sensors for the duration of the study. Sensor data is a useful research tool and studies that have investigated sensor data have shown improvements in healthy lifestyle outcomes particularly related to managing weight, increasing physical activity, and improving sedentary behaviors [35,36] although these did not focus on stress and burnout among nursing students.

Recommendations for future studies include: Looking into the impact of NBI on stress levels of recently graduated nurses who are practicing in the field and caring for patients; configuring the survey to be more user friendly, along with clarifying any survey question wording; conducting interviews with participants either individually or in focus groups to gain a greater perspective on how they define nature and what it means to them at the start and end of the study; and incentives to encourage participants to complete all study requirements. In the future, we would like to incorporate a saliva cortisol test to further assess the effects of increased nature exposure on stress levels.

## 5. Limitations

This feasibility pilot limitations should be noted when reviewing the results. First, as a feasibility study, the small sample size limits generalizability of the study We did not ask participants what their usual nature activity was at baseline nor the quality of nature they frequented. Additional stressful events during the course of this study was beyond the scope of data collection.

## 6. Conclusions

The results of this study reinforce the feasibility of NBI for undergraduate nursing students. Regarding feasibility of our participants, 100% wore their electronic sensor for the duration the study. This study’s results contribute to maximizing the opportunities for feasible, affordable, and sustainable interventions to reduce stress among undergraduate nursing students. This was an important topic before the COVID-19 pandemic however, COVID-19 has exaggerated the stress that many nurses encounter. This is vital research to determine feasible interventions support our nursing students, the future of nurses’ wellbeing, and their ability to continue caring for patients. This feasibility study is considered a bridge to a more extensive study to develop national guidelines for nature therapy enhancing the wellbeing of healthcare providers. With greater intention of being exposed to nature, there were more positive results regarding the quality of life of the participant.

Recommendations to mitigate burnout includes promotion of health, wellbeing, and self-care first at the individual level, and then, subsequently, at the institutional level [37]. Participating in NBI promotes a sustainable and feasible approach to positively affect the health and wellbeing of undergraduate nursing students and recently graduated registered nurses (RNs). This feasibility study informs our next steps in planning for a nature-based intervention study. The long-term goal of this project is to develop national guidelines for nature therapy to enhance the wellbeing of healthcare providers.

## Figures and Tables

**Table 1:** Electronic sensor measures over time.

**HR (in BPM)**	**Week 1**	**Week 2**	**Week 3**	**Week 4**
CONTROL AVERAGE	76.9	75.7	77.6	76.3
INTERVENTION AVERAGE	77.0	75.6	75.9	75.6
**Daily Steps**	**Week 1**	**Week 2**	**Week 3**	**Week 4**
CONTROL AVERAGE	8716.4	8806.7	8942.4	8453.3
INTERVENTION AVERAGE	8259.7	7455.6	7896.7	7884.0
**Daily Sleep (in hours)**	**Week 1**	**Week 2**	**Week 3**	**Week 4**
CONTROL AVERAGE	7.54	7.58	7.27	7.19
INTERVENTION AVERAGE	7.53	7.82	7.89	7.58

## Data Availability

Not applicable.
